# Molecular Mechanisms of PALB2 Function and Its Role in Breast Cancer Management

**DOI:** 10.3389/fonc.2020.00301

**Published:** 2020-02-28

**Authors:** Shijie Wu, Jiaojiao Zhou, Kun Zhang, Huihui Chen, Meng Luo, Yuexin Lu, Yuting Sun, Yiding Chen

**Affiliations:** ^1^Department of Breast Surgery, The Second Affiliated Hospital, Zhejiang University School of Medicine, Hangzhou, China; ^2^The Key Laboratory of Cancer Prevention and Intervention, China National Ministry of Education, Zhejiang University School of Medicine, Hangzhou, China

**Keywords:** PALB2, homologous recombination, breast cancer, precision medicine, pathogenic variants

## Abstract

Partner and localizer of BRCA2 (PALB2) is vital for homologous recombination (HR) repair in response to DNA double-strand breaks (DSBs). PALB2 functions as a tumor suppressor and participates in the maintenance of genome integrity. In this review, we summarize the current knowledge of the biological roles of the multifaceted PALB2 protein and of its regulation. Moreover, we describe the link between *PALB2* pathogenic variants (PVs) and breast cancer predisposition, aggressive clinicopathological features, and adverse clinical prognosis. We also refer to both the opportunities and challenges that the identification of *PALB2* PVs provides in breast cancer genetic counseling and precision medicine.

## Introduction

Partner and localizer of BRCA2 (PALB2) is encoded on chromosome 16p12.2 and comprises 1186 residues (1). As a major BRCA2 binding partner, PALB2 licenses the function of BRCA2 and participates in homologous recombination (HR), a faithful DNA double-strand break (DSB) repair pathway in mammalian cells (2–4). Numerous studies have demonstrated that biallelic mutations in *PALB2* resulted in a subtype of Fanconi anemia (FA-N), while monoallelic *PALB2* mutations predispose carriers to multiple cancers such as breast, pancreatic, and ovarian cancers (5–8).

Breast cancer is the most frequently diagnosed cancer and the major cause of cancer death among women worldwide (9). Approximately 10–15% of breast cancer cases are due to familial and genetic factors, underscoring the great significance of genetic susceptibility in breast cancer development (10). Previous studies have identified a broad range of breast cancer susceptibility genes, including *BRCA1, BRCA2*, and *TP53* (11). However, the high-penetrance *BRCA1* and *BRCA2* are responsible for only ~20% of the familial aggregation of breast cancer (12, 13), and syndromic breast cancer susceptibility genes such as *TP53, PTEN*, and *CDH1* are estimated to explain just 5% of familial breast cancers (14). Large-scale analyses of multigene panel testing recently confirmed *PALB2* as a high-risk breast cancer susceptibility gene (15), and the odds ratio (OR) of *PALB2* mutations for breast cancer was comparable to that of *BRCA2* mutations (16). Hence, a comprehensive understanding of the biological functions of PALB2 is vital for breast cancer management and precision medicine.

## Structures of PALB2 and Its Biological Functions in HR

**Figure 1:**
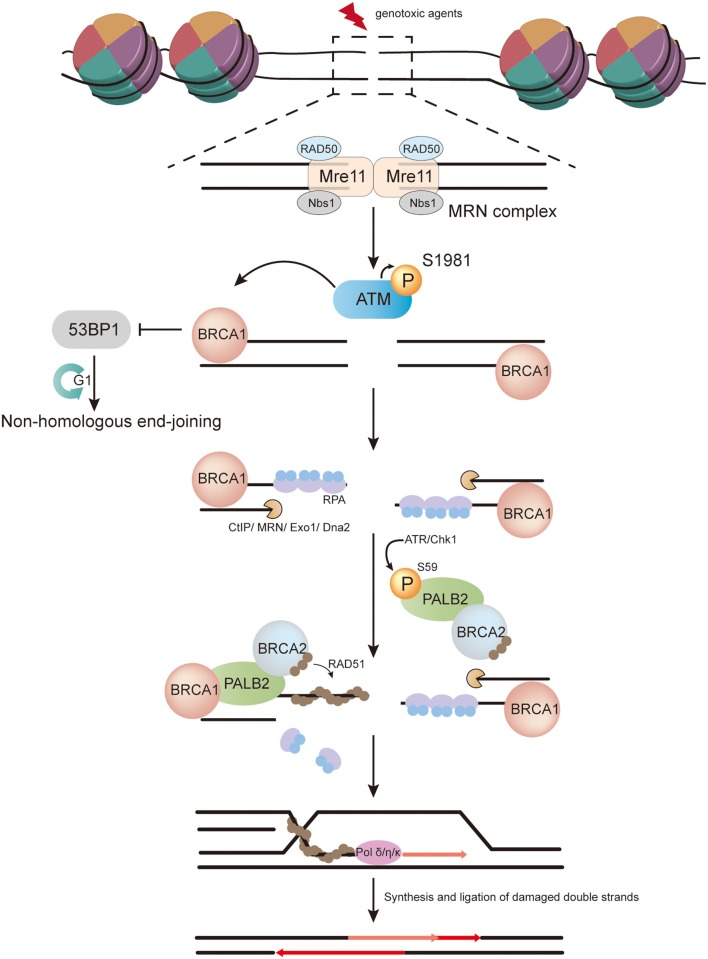
The role of PALB2 in homologous recombination (HR). In response to DNA double-strand breaks (DSBs) induced by genotoxic agents in the S/G2 phase, the Mre11–RAD50–Nbs1 (MRN) complex is recruited to DSBs and promotes ATM recruitment. The inactive ATM dimer then dissociates into active monomers through autophosphorylation at serine 1981. Active ATM monomers phosphorylate H2AX in regions of DSBs and create a platform to recruit BRCA1, which facilitates a shift from non-homologous end-joining to HR. Meanwhile, CtBP-interacting protein (CtIP), in conjunction with the MRN complex, catalyzes 5′-3′ resection at DSBs to generate single-stranded DNA (ssDNA), and further resection is completed by Exo1 exonuclease and Dna2 nuclease/helicase in cooperation with BLM helicase. The resulting ssDNA is then covered by replication protein A (RPA). PALB2 is phosphorylated on S59 by ATR/Chk1, which accelerates its recruitment to sites of damage. Thereafter, BRCA2 is recruited by PALB2. PALB2 and BRCA2 further promote RPA removal and RAD51 loading. The resulting RAD51-ssDNA filament invades the intact sister chromatid and extends the strand with the help of DNA polymerase δ/η/κ. Finally, further restoration and ligation of double strands are carried out.

**Figure 2:**
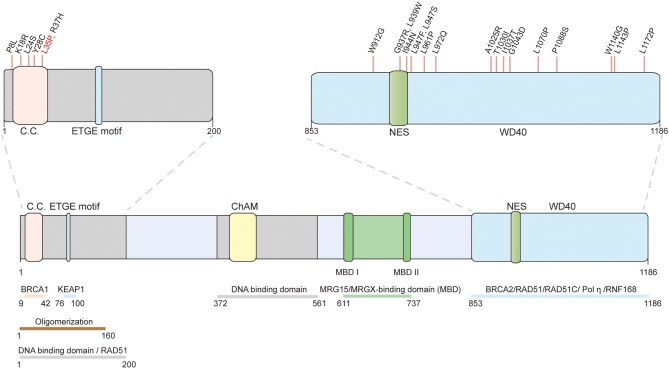
Schematic representation of the PALB2 protein and the position of functionally validated *PALB2* pathogenic missense variants. The structural motifs and functional domains of PALB2. C.C.: coiled-coil motif (9–42); ETGE motif (88–94); ChAM: chromatin-association motif (395–446); WD40: WD40-repeats (853–1186); NES: nuclear export sequence (928–945). The validated pathogenic missense variants are marked on top. The only recognized *PALB2* pathogenic missense variant (p.L35P) validated by systematic *in vitro* functional assays is highlighted in red.

PALB2, first described by Xia et al. in 2006 (1), has an important role in HR. It mainly serves as a bridging molecule that connects the BRCA complex (BRCA1-PALB2-BRCA2-RAD51) and facilitates the function of RAD51, a protein vital for strand invasion during HR (Figure 1). The role of PALB2 in HR has been shown to involve several protein domains, including a coiled-coil domain, a WD40 domain, and a chromatin-association motif (ChAM) (Figure 2).

The coiled-coil domain is located in the N terminus of PALB2 (residues 9–42) and is responsible for its interaction with BRCA1 (2–4). The L21A, Y28A, and L35A mutations in the PALB2 coiled-coil domain disrupt the BRCA1-PALB2 interaction, impairing the function of PALB2 in HR repair and inducing hypersensitivity to mitomycin C (MMC) treatment (3). In addition to positively regulating HR, the BRCA1-PALB2 interaction is required for preventing single-strand annealing (SSA), which is a deletion-causing DSB repair pathway. Using U2OS/DR-GFP and U2OS/SA-GFP reporter cells, Anantha et al. demonstrated that depletion of either PALB2 or BRCA2 led to impaired HR activity and a substantial increase in SSA, whereas BRCA1 depletion caused a reduction of both HR and SSA activity (17). These results established that BRCA1 is essential for DSB repair, while PALB2 serves to direct the DSB repair toward the HR pathway following resection.

The WD40 domain is located in the PALB2 C-terminus and in the shape of a WD40-type β-propeller with seven blades (18). This domain is involved in the interaction with BRCA2, DNA polymerase η, RAD51, RAD51C, and the ubiquitin ligase RNF168 (5, 19–21). Even a single nucleotide change within the WD40 region (e.g., L939W) can disturb the PALB2-BRCA2 interaction and causes HR deficiency (20). The WD40 domain of PALB2 is also crucial for the interaction with DNA polymerase η, which is vital for the initiation of HR-mediated DNA synthesis and D-loop extension (19). Recently, a hidden nuclear export sequence (NES) was found in the WD40 domain of PALB2. The breast cancer-associated *PALB2* truncating mutation, W1038X, exposes this NES, resulting in PALB2 translocation to the cytoplasm and defects in HR (22).

The ChAM is an evolutionarily conserved domain located in the middle region of PALB2 (23). ChAM-deleted PALB2 has a compromised role in supporting MMC-induced RAD51 focus formation, suggesting that ChAM promotes the function of PALB2 through chromatin association (23). The ChAM binds to nucleosomes and participates in the formation of the PALB2-BRCA2-RAD51 complex on chromatin, which rapidly transforms into an active BRCA complex following DSBs (23).

In addition to BRCA complex formation, PALB2 also directly interacts with RAD51 and enhances its strand invasion activity (24, 25). *In vitro* D-loop assays revealed increased product formation when PALB2 was included in the RAD51 reaction. Moreover, Buisson et al. also identified two DNA-binding domains in PALB2 (24) (Figure 2). More recently, Deveryshetty et al. (26) showed that the main DNA-binding domain (DBD) of PALB2 is located in its N-terminus (N-DBD, residues 1–200). Mutation of just four amino acids in the N-DBD significantly disrupts the HR activity of PALB2. Surprisingly, the authors discovered that the N-DBD of PALB2 enhances RAD51-mediated strand exchange and also promotes a similar reaction in the absence of RAD51. Using strand exchange fluorescent assays, they further demonstrated that PALB2 N-DBD promotes both forward and inverse strand exchange using either DNA or RNA as substrate (26).

These studies uncovered multiple effects of PALB2 during HR. On the one hand, PALB2 serves as the bridging molecule in the BRCA complex; on the other hand, it potently stimulates strand invasion in HR.

## PALB2: A Versatile Player in Biological Regulation

### PALB2 and Chromatin Association

**Figure 3:**
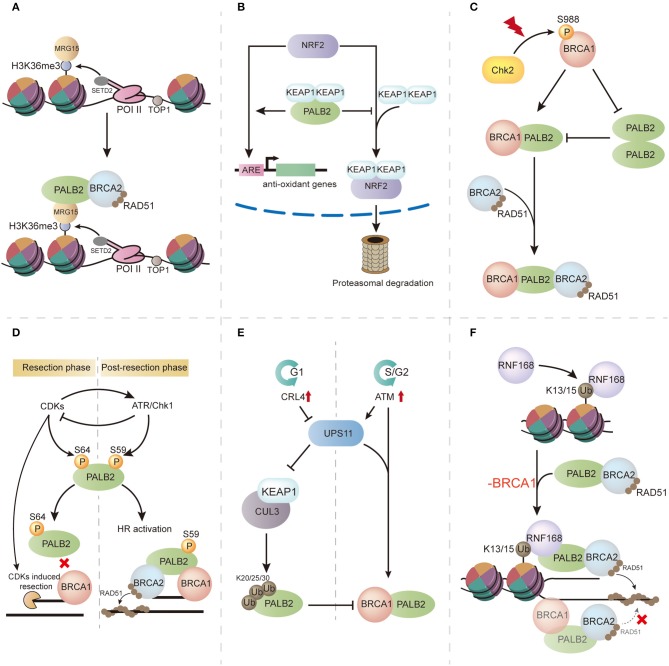
The multifaceted functions of PALB2 and its regulation. **(A)** PALB2 is recruited through the SETD2/H3K36me3/MRG15 axis and protects transcriptionally active genes from replication stress. **(B)** PALB2 promotes NRF2 function during oxidative stress by competitively binding KEAP1. **(C)** Following ionizing radiation (IR), the switch from PALB2 oligomerization to BRCA1-PALB2 interaction is regulated by S988 phosphorylated BRCA1. **(D)** Phosphorylation events in PALB2 regulation. In the resection phase, high CDKs induce PALB2 phosphorylation at S64, inhibiting its interaction with BRCA1, whereas in post-resection phase, ATR-induced PALB2 phosphorylation at S59 promotes BRCA1-PALB2 binding and enhances HR activity. **(E)** In the G1 phase of the cell cycle, PALB2 is ubiquitylated by the CUL3–KEAP1 complex, which disrupts BRCA1-PALB2 interaction, whereas in the G2 phase, PALB2 ubiquitylation is neutralized by USP11. **(F)** RNF168 mediates PALB2 recruitment and RAD51 loading in BRCA1-deficient cells.

Chromatin association is considered indispensable for the biological function of PALB2. In addition to the ChAM, MRG15 is another PALB2-interacting factor involved in PALB2 chromatin association (27) (Figure 3A). In 2009, Sy et al. unveiled MRG15 and another MORF-related gene product, MRGX, as PALB2 cooperators through tandem affinity purification and mass spectrometry analysis (28). MRG15 belongs to the highly conserved MRG protein family (29) and has two functional domains: one is an MRG domain that binds to PALB2 as well as multiple transcriptional regulators (28, 30), the other is N-terminal chromodomain that binds lysine 36-trimethylated histone H3 (H3K36me3) (31), which is mediated by lysine methyltransferase SET domain containing 2 (SETD2) (32). The MRG15-binding region was roughly mapped to the middle region of PALB2 (residues 611–764) and exactly matched two highly conserved regions named MBD-I (residues 611–629) and MBD-II (residues 724–737) (33). Sy et al. found that reconstitution of MRG15-binding domain deleted PALB2 could restore RAD51 foci formation and cell survival after MMC treatment in EUFA1341F PALB2-deficient cells (28). Strikingly, a concurrent study reached a contradictory conclusion, whereby siRNA-mediated MRG15 depletion in cells compromised HR repair efficiency and sensitization to MMC (34). Furthermore, MRG15 knockout murine embryonic fibroblasts exhibited moderate sensitivity to γ-irradiation and decreased capacity for RAD51 nuclear foci formation (35). Considering the studies above, we could propose that the MRG15-PALB2 interaction is involved in HR repair, but may not be critical for this process.

Intriguingly, a genome-wide analysis evaluating PALB2 chromatin residence revealed a tight relationship between PALB2 chromatin residence and transcriptionally active genes (36). This result confirmed that MRG15-PALB2 interaction is associated with unperturbed chromatin. In 2017, Bleuyard et al. hypothesized the innovative concept that the MRG15-PALB2 interaction within undamaged chromatin maintains chromatin stability during DNA replication (33) (Figure 3A). This idea was supported by genome-wide PALB2 chromatin immunoprecipitation-sequencing analysis, which indicated gathering of PALB2 at H3K36me3-modified genes through the SETD2/H3K36me3/MRG15 axis. Moreover, expression of MRG15 binding-defective PALB2 leads to compromised proliferation, DNA stress, and genome instability when compared with wild-type PALB2 expression in EUFA1341 cells (33). These findings indicate that the MRG15-PALB2 complex may be a genomic stabilizer within active genes, which renders PALB2 immediately available following DNA damage and guarantees a rapid response to replication stress, thereby maintaining genome stability. In addition to MRG15-PALB2 interaction, PALB2 is also recruited by phosphorylated replication protein A (RPA) during replication stress. Murphy et al. revealed that phosphorylation of RPA during replication stress stimulates the recruitment of PALB2 and increases the stability of PALB2 chromatin binding, making PALB2 available to alleviate replication stress and facilitating the recovery of stalled replication forks (37).

### PALB2 and Oxidative Stress

KEAP1, an oxidative stress mediator that negatively regulates the function of the antioxidant transcription factor NRF2, was revealed to bind PALB2 by coimmunoprecipitation (38). Surprisingly, a highly conserved 7-aa motif (LDEETGE) within the KEAP1 binding domain of PALB2 (residues 76–100) was identical to the ETGE motif of NRF2 that binds KEAP1, implying that PALB2 may promote the role of NRF2 by competitively binding KEAP1 (Figure 3B). This was supported by increased levels of reactive oxygen species (ROS) and reduced expression of NRF2-regulated genes after PALB2 depletion. Thus, this study unveiled a unique function of PALB2 during oxidative stress and provided a possible link between the oxidative stress response and PALB2-associated cancer formation.

### PALB2 and Cell-Cycle Checkpoint Control

Cell-cycle checkpoints are essential for DNA damage repair following genotoxic exposure because of their role in constraining cell-cycle progression and providing time for accurate DSB repair by HR, thereby guaranteeing genome stability (39). Menzel et al. performed a high-throughput siRNA screen to explore potential G2 checkpoint modulators, and identified PALB2 as a main G2 checkpoint maintainer (40). Depletion of PALB2 led to G2 checkpoint dysregulation and premature checkpoint recovery. In the same study, the role of PALB2 in the maintenance of the G2 checkpoint was seemly independent of the HR pathway, as RAD51 depletion did not compromise G2 checkpoint control. More recently, Simhadri et al. proposed a novel model in which PALB2 serves as a nexus that connects BRCA1 and BRCA2 in G2/M checkpoint control (41). Consistent with this view, disturbing the interactions of BRCA1-PALB2 or BRCA2-PALB2, using the L35P or A1025R mutant of PALB2, respectively, severely impaired the checkpoint response. Notably, BRCA1-PALB2 interaction seems to be critical for checkpoint initiation, whereas BRCA2-PALB2 interaction plays a more significant role in checkpoint maintenance. Although these studies have unveiled the role of PALB2 in checkpoint control, it remains unclear how exactly PALB2 participates in the pathway.

## Regulation of PALB2

The biological functions of PALB2 are strictly regulated. To date, many mechanisms of its regulation have been elucidated, including PALB2 oligomerization, phosphorylation, ubiquitylation, and interaction with RNF168.

### PALB2 Oligomerization

PALB2 oligomerization negatively regulates HR through its coiled-coil domain (42, 43) (Figure 3C). Overexpression of the PALB2 coiled-coil domain markedly impairs RAD51 filament formation, suggestive of competition between PALB2 oligomerization and BRCA1-PALB2 interaction. Meanwhile, immunoprecipitation analyses showed that the presence of BRCA1 completely abrogated PALB2 self-interaction, indicating that PALB2 self-interaction can be inhibited by its interaction with BRCA1 (43). As the Chk2-induced BRCA1 phosphorylation of S988 is important for HR activity (44), Buisson et al. proposed that BRCA1 phosphorylation may lead to a molecular switch from PALB2 homodimerization to BRCA1-PALB2 interaction, thereby promoting HR (43) (Figure 3C). Song et al. recently reported that PALB2 homodimerization is mediated by an antiparallel coiled-coil leucine zipper (45). Mutation of residue Leu24, a key stabilizer at the dimer interface, greatly reduces PALB2 homodimer stability and results in genomic instability in mutated cells, suggesting an important role of PALB2 oligomerization in HR regulation.

### PALB2 Phosphorylation

PALB2 phosphorylation is also critical for its modulation. Three N-terminal S/Q sites of PALB2 (S59, S157, and S376) were found to be phosphorylated following ionizing radiation, and the phosphorylation events were mediated by ataxia telangiectasia mutated protein (ATM) and ATM and Rad3-related kinase (ATR) (46, 47). Phosphorylation-deficient PALB2 failed to promote RAD51 foci formation, leading to impaired HR and genome instability, highlighting the role of phosphorylation in PALB2 regulation (47). Strikingly, Buisson et al. (48) demonstrated a phosphorylation conversion at S59 and S64 on PALB2 during the phosphorylation process (Figure 3D). In this model, PALB2 is first phosphorylated at S64, a cyclin-dependent kinase (CDK) site, and high CDK activity actuates DNA end resection and ATR activation. Activated ATR then induces S59 phosphorylation and suppresses CDK activity, followed by hypo-phosphorylation of S64 and a strengthened BRCA1-PALB2 interaction (48). This CDK-ATR switch is crucial for attaining optimal levels of PALB2 at DSBs.

### PALB2 Ubiquitylation

Ubiquitylation has also been reported to regulate PALB2 function via cell-cycle control (Figure 3E). In the G1 phase, the CUL3–KEAP1 complex ubiquitylates PALB2 on its N terminus, which is the BRCA1-binding region, to suppress BRCA1-PALB2 interaction, ultimately inhibiting HR. As cells enter the S/G2 phase, PALB2 ubiquitylation is neutralized by USP11, a deubiquitylase that is antagonized by CRL4 in the G1 phase. Restoration of BRCA1-PALB2 interaction facilitates BRCA complex formation and induces HR repair (49).

### PALB2 and RNF168

The E3 ubiquitin ligase RNF168 was recently found to promote PALB2 accumulation in the S/G2 phase and facilitate DNA repair. It was supported by the restoration of PALB2 foci in endogenous RNF168-depleted S/G2 cells after re-expression of RNF168. The intrinsic mechanism is a physical interaction between the WD40 domain of PALB2 and the newly uncovered PALB2-interacting domain of RNF168 (21). Recently, Zong et al. revealed that RNF168-driven PALB2 recruitment, a BRCA1-independent pathway, serves as a backup to maintain DNA repair by HR (50) (Figure 3F). In this model, PALB2 recruitment is mainly orchestrated by BRCA1 in BRCA1-proficient cells, and an RNF168-driven pattern is applied as an auxiliary. Nevertheless, in *BRCA1* mutated cells, RNF168-mediated PALB2 recruitment plays a vital alternative role for RAD51 loading and genome stability. Considering the unambiguous association between RNF168 and PALB2, inhibiting RNF168 signaling in BRCA1-insufficient cancers may be an effective therapeutic strategy (51).

## PALB2 and Diseases

### PALB2 and Fanconi Anemia

Fanconi anemia (FA) is a rare human genetic instability syndrome associated with diverse developmental defects, early-onset bone marrow failure, and cancer predisposition, mainly to acute myeloid leukemia and head and neck squamous cell carcinoma. Cells derived from FA patients are hypersensitive to DNA crosslinking agents such as MMC and cisplatin, and this hallmark is commonly used for the clinical diagnosis of FA (52). To date, 22 FA-related proteins have been identified in the FA-BRCA pathway for DNA interstrand cross-link repair (53–55), and PALB2 serves as a mediator in the BRCA pathway (56). In 2007, Xia et al. (5) reported a new subtype of Fanconi anemia (FA-N) resulted from biallelic mutations in *PALB2* (also known as *FANCN*). A PALB2-deficient Fanconi anemia cell line showed impaired RAD51 foci formation and hypersensitivity to MMC treatment (5). Notably, FA-N patients are at a high risk of developing embryonal cancer, similar to that seen in patients with biallelic *BRCA2* mutations, but differing from that observed for patients of other FA subtypes (57). These findings emphasize the important role of PALB2 in maintaining genomic stability and tumor suppression.

### PALB2 and Breast Cancer

PALB2 is tightly correlated with breast cancer and has been associated with breast cancer predisposition, clinicopathological features, and prognosis.

In 2007, Rahman et al. (6) provided a profile of *PALB2* mutations in breast cancer predisposition. The authors determined the frequency of *PALB2* monoallelic truncating variants in a familial breast cancer cohort negative for *BRCA* mutations (10/923, 1.1%), which was far more common than in controls (0/1,084, 0%; *p* = 0.0004). They also revealed that individuals with monoallelic *PALB2*-mutations had a 2.3-fold increased risk of breast cancer compared with controls (95% confidence interval [CI], 1.4–3.9; *p* = 0.0025) (6), hinting that monoallelic *PALB2* mutations may have a more moderate role in breast cancer predisposition than monoallelic *BRCA2* variants (58, 59). At the same time, research in Finland identified a recurrent mutation, c.1592delT, in 1% of unselected breast cancer patients (60). This frameshift mutation resulted in a 40% cumulative risk of developing breast cancer by age 70 (95% CI, 17–77) (61), similar to that for *BRCA2* mutation carriers (~45%; 95% CI, 31–56) (62), implying a striking role of *PALB2* in predisposition to breast cancer. Subsequently, multiple population-based screenings of *PALB2*-truncating mutations reported 2–30-fold increases in breast cancer risk for *PALB2*-truncating mutations carriers (6, 63–67). In 2014, Antoniou et al. (64) estimated the age-specific relative risk for *PALB2* mutation carriers, which was highest among women before age 40 years (relative risk, 8–9) then gradually declined with age, with the lowest risk after the age of 60 years (relative risk, ~5). Meanwhile, female *PALB2* mutation carriers showed an estimated cumulative breast cancer risk of 35% (95% CI, 26–46) by age 70 (64). A recent international study from 21 countries that comprised 524 families with *PALB2* pathogenic variants (PVs) revealed that the estimated relative risk associated with *PALB2* PVs for breast cancer in females was 7.18 (95% CI, 5.82–8.85; *p* = 6.5 × 10^−76^). The authors also showed that the estimated relative risk for female breast cancer declined with age, varying from 13.1 at young ages to 4.69 for older ages, and the estimated female breast cancer risk was 53% (95% CI, 44–63) to age 80 years (68).

Male breast cancer (MBC) is a rare disease that accounts for <1% of all breast cancer cases (69). However, ~20% of MBC patients have a family history of breast cancer (70), highlighting a strong correlation between genetic susceptibility genes and MBC. Two high-penetrance breast cancer genes, *BRCA1* and *BRCA2*, are thought to be responsible for only 13% of MBC (71), and multiple genetic factors remain unknown. To date, many PVs of *PALB2* have been reported in MBC patients (72–76). In 2017, Pritzlaff et al. uncovered that *PALB2* variants significantly increased the risk of MBC (OR, 6.6; *p* = 0.01) (77). This viewpoint was further supported by an Italian population-based multicenter study in 2019 (78). Rizzolo et al. found that *PALB2* was the most frequently mutated gene (1.2%) among non-BRCA1/2 altered MBC patients, and deleterious *PALB2* variants conferred a 9.63- to 17.30-fold increased risk of MBC (78). More recently, Yang et al. further showed an estimated MBC relative risk of 7.34 (95% CI, 1.28–42.18; *p* = 0.026) for *PALB2* PVs carriers by analyzing data from 524 families with *PALB2* PVs from 21 countries (68).

Several studies have also found that *PALB2*-mutated breast cancer is associated with aggressive clinicopathological features. In 2009, Heikkinen et al. reported that breast cancer patients harboring the *PALB2* c.1592delT mutation were more likely to present the triple-negative phenotype (54.5%, *p* < 0.0001), characterized by the absent expression of estrogen receptor, progesterone receptor, and human epidermal growth factor receptor 2 (79), than other familial (12.2%) or sporadic (9.4%) breast cancer patients (80). This finding was further supported by other population-based screening studies (10, 81–84). Heikkinen et al. also showed that *PALB2*-mutated breast cancer patients were more likely to present at an advanced disease stage (*p* = 0.0027 and *p* = 0.0017, respectively) and have a higher Ki67 level (*p* = 0.0004 and *p* = 0.0490, respectively) compared with other familial or sporadic patients (80).

In 2015, Cybulski et al. first evaluated the prognostic effects of two *PALB2* deleterious mutations (509_510delGA and 172_175delTTGT) in Poland (83). In this study, the 10-year survival rate for female breast cancer patients with a *PALB2* mutation was 48.0% (95% CI, 36.5–63.2), significantly lower than that for *PALB2* mutation-negative female breast cancer patients (74.7%; 95% CI, 73.5–75.8). The 10-year adjusted hazard ratio for all-cause mortality was 2.27 (95% CI, 1.64–3.15; *p* < 0.0001), indicating an adverse prognostic effect of *PALB2* in breast cancer (83). More recently, a population-based screening of breast cancer susceptibility genes in China further confirmed the prognostic value of *PALB2* in breast cancer; patients with a *PALB2* mutation presented shorter overall survival compared with noncarriers (adjusted hazard ratio, 8.38; 95% CI, 2.19–32.11; *p* = 0.002) (85).

### PALB2 and Other Cancers

In addition to breast cancer, *PALB2* has also been identified as a susceptibility gene for pancreatic cancer. Jones et al. (86) first discovered a germline *PALB2*-truncating mutation (c.172_175delTTGT) in a familial pancreatic cancer (FPC) patient, and three *PALB2*-truncating mutations were further identified in 96 additional FPC patients (3.1%). In contrast, no *PALB2*-truncating mutation was found in 1,084 normal individuals (86). Subsequent studies also revealed the prevalence of *PALB2* deleterious mutations in patients with FPC (~3–4%) (87, 88), validating the role of *PALB2* mutations in pancreatic cancer predisposition. To date, several studies have indicated that *PALB2* mutations are associated with ovarian cancer (8, 89, 90). Although the mutation frequency was low, Norquist et al. demonstrated that *PALB2* mutation carriers had a significantly higher risk of ovarian cancer compared with the NHLBI Exome Sequencing Project (OR, 10.2; 95% CI, 2.2–47.0; *p* < 0.001) or the Exome Aggregation Consortium database (OR, 4.4; 95% CI, 2.1–9.1; *p* < 0.001) (8). Pathogenic *PALB2* mutations have also been identified in patients with other cancers, such as gastric and prostate cancer; however, whether these mutations confer an increased cancer risk for these cancer types requires further research (91–94).

## PALB2 and Precision Medicine

Excluding *BRCA1/2*, PVs in *PALB2* contribute most significantly to the mutation detection rate in multigene testing panels for hereditary breast cancer (95). Thus, germline *PALB2* status is crucial for breast cancer risk assessment in individuals with an apparent family history of breast cancer. To date, several ethnic-specific *PALB2* recurrent mutations have been reported, and related cancer predisposition risks have been established in distinct territories (60, 63, 82, 83, 96, 97) (Table 1). In these regions, genotyping for specific *PALB2* PVs can be applied as a cost-effective tactic in high-risk individuals. For most high-risk people who are not in these specific regions, multigene panel testing that includes *PALB2* is an advisable choice for genetic counseling (98–100). According to National Comprehensive Cancer Network (NCCN) guidelines, annual mammogram with consideration of tomosynthesis and breast magnetic resonance imaging with contrast are recommended for people with *PALB2* PVs/likely PVs from the age of 30 to detect cancer at an early stage (101).

**Table 1:** Ethnic-specific *PALB2* recurrent mutations and related breast cancer predisposition.

**Exon**	**Nucleotide change**	**Protein effect**	**Ethnicity**	**Familial breast cancer**		**Cancer** **predisposition** **(95% confidence interval;** ***p*-value)**	**Unselected breast cancer**		**Cancer** **predisposition** **(95% confidence interval;** ***p*-value)**	**References**
				**Carriers/total probands studied**	**Controls**		**Carriers/total probands studied**	**Controls**		
Ex4	c.1592delT	L531fs	Finnish	3/113 (2.7%)	6/2,501 (0.2%)	Odds ratio: 11.3 (1.8–57.8; *p* = 0.005)	18/1,918 (0.9%)	6/2,501 (0.2%)	Odds ratio: 3.94 (1.5–12.1; *p* = 0.003)	(60)
Ex4	c.509_510delGA	R170fs	Polish	4/648 (0.6%)	1/1,310 (0.08%)	– (–; *p* = 0.044)	–	–	–	(82)
Ex4	c.509_510delGA	R170fs	Polish	–	–	–	76/12,529 (0.61%)	7/4,702 (0.15%)	Odds ratio: 4.09 (1.89–8.88; *p* < 0.0001)	(83)
Ex4	c.1027C>T	Q343X	Italian (Bergamo)	6/113 (5.3%)	2/477 (0.4%)	– (–; *p* < 0.01)	–	–	–	(97)
Ex5	c.2323C>T	Q775X	French-Canadian	–	–	–	2/356 (0.56%)	0/6,440 (0%)	– (3.4–∞; *p* = 0.0027)	(96)
Ex10	c.3113G>A	W1038X	Australian	8 /779 (1%)	0/764 (0%)	–	5/1,403 (0.4%)	0/764 (0%)	Hazard ratio: 30.1 (7.5–120; *p* < 0.0001)	(63)

Poly (ADP-ribose) polymerases (PARPs) act as DNA damage sensors and regulators and play important roles in the repair of single-stranded DNA breaks through the base-excision repair pathway (102). PARP inhibitor (PARPi) treatment prevents the repair of single-stranded DNA breaks and leads to DSBs in cells. *BRCA1/2*-deficient cells are unable to repair DSBs via the HR pathway, resulting in cell death (103). Consequently, PARP inhibition is considered a promising strategy for the treatment of *BRCA1/2*-deficient tumors through synthetic lethality. More recently, the PARPi olaparib and talazoparib have been approved for germline BRCA-mutated (gBRCAm) HER2-negative metastatic breast cancer in clinic (104). Similar to *BRCA1/2, PALB2* is an essential component in HR-based DNA repair, and *PALB2* loss of function was shown to be synthetic lethal in combination with PARPi (105). Recently, PARPi sensitivity of *PALB2* missense variants has been partially elucidated *in vitro*. Foo et al. identified a PARPi-hypersensitive *PALB2* variant (p.L35P) using EUFA1341 cells, an FA-N patient-derived skin fibroblast cell line with biallelic mutations in *PALB2* (106). In 2019, Rodrigue et al. (107) utilized siRNA-mediated RNA interference to generate PALB2-depleted HeLa cells, and exogenous siRNA-resistant PALB2 variants were complemented before PARPi sensitivity assay. Cells expressing the *PALB2* variants p.T1030I or p.W1140G showed significantly higher olaparib sensitivity than those expressing wild-type *PALB2* (107). A concurrent study conducted by Wiltshire et al. revealed four new PARPi-hypersensitive variants in *PALB2* (p.L24S, p.I944N, p.A1025R, and p.L1070P) using PALB2-deficient B400 mouse mammary tumor cells (108). Boonen et al. (109) developed a cDNA-based system for the functional analysis of *PALB2* variants. By evaluating the ability of *PALB2* variants to rescue PARPi sensitivity in *PALB2* knockout mouse embryonic stem cells, they identified twelve *PALB2* variants (p.Y28C, p.L35P, p.W912G, p.G937R, p.I944N, p.L947S, p.L961P, p.L972Q, p.A1025R, p.T1030I, p.G1043D, and p.L1172P) that showed hypersensitivity to PARPi (109).

In spite of the lack of clinical evidence for PARPi treatment efficacy in *PALB2*-deficient breast cancer patients, the response of some other *PALB2*-deficient solid tumors to PARPi in clinical/preclinical studies have been remarkable. A preclinical study of BMN673 (talazoparib) for the panel of the Pediatric Preclinical Testing Program showed that a maintained complete response was observed *in vivo* in a Wilms tumor xenograft model, characterized by a truncating mutation in *PALB2* (p.Y1108fs) (110). de Bono et al. (111) conducted a phase I trial of the PARPi talazoparib in patients with advanced solid tumors, and reported an objective response rate of 20% in 10 pancreatic cancer patients treated with 1.0 mg/day talazoparib. Of the two patients who showed a partial response, one harbored a mutation in *BRCA2*, and the other harbored a mutation in *PALB2* (111). These results suggest that PARPi exerts a synthetic lethal effect in *PALB2*-deficient tumors. Several clinical trials of PARPi are currently in progress for breast cancer with mutations in DNA repair genes, including *PALB2*. A phase II trial is underway for the evaluation of PARPi olaparib monotherapy in the treatment of metastatic breast cancer patients harboring germline/somatic mutations in non-*BRCA1/BRCA2* DNA repair genes (NCT03344965). A different phase II clinical trial of the PARPi talazoparib is being performed for non-*BRCA1/BRCA2*-mutated patients with advanced triple-negative breast cancer and HR deficiency or advanced HER2-negative solid tumors harboring a germline/somatic mutation in a HR pathway gene, such as *PALB2* (NCT02401347). The outcomes of these trials are expected to expand the potential applications for PARPi therapy.

Overall, these data indicate that *PALB2* status should be assessed and included in genetic counseling and patient treatment regimens for best clinical outcome.

## The Challenges of PALB2 Research in Clinical Application

Population-based screening has identified numerous *PALB2* variants, and the frequency-penetrance profiles of some ethnic-specific *PALB2* PVs have been described. At least 604 distinct variants in *PALB2* have been discovered according to an established database (https://databases.lovd.nl/shared/variants/PALB2/unique); however, only ~140 of the variants are thought to be pathogenic, whereas more than 400 are missense variants of unknown significance (VUSs). The lack of verification toward these VUSs challenges genetic counseling (27). Here, we summarize the breast cancer-associated missense variants of *PALB2* that have been functionally verified (Figure 2). Pathogenic *PALB2* missense variants are mainly located in the N- and C-terminus. In the PALB2 N-terminus, p.L35P (c.104T>C) disrupts BRCA1-PALB2 interaction and abolishes the HR activity of PALB2, resulting in sensitivity to the PARPi (106). Moreover, p.P8L (c.23C>T), p.K18R (c.53A>G), p.L24S (c.71T>C), p.Y28C (c.83A>G), and p.R37H (c.110G>A) compromise HR activity of PALB2 and are suggested to be pathogenic (106–108). In the PALB2 C-terminus, p.W912G, p.G937R, p.L939W, p.I944N (c.2831T>A), p.L947F (c.2841G>T), p.L947S (c.2840T>C), p.L961P, p.L972Q, p.A1025R, p.T1030I (c.3089C>T), p.I1037T, p.G1043D, p.L1070P (c.3209T>C), p.P1088S (c.3262C>T), p.W1140G (c.3418T>C), p.L1143P, and p.L1172P promoted a decrease in the HR activity of PALB2 (20, 107–109, 112). Among these mutations, p.L939W, p.A1025R, p.T1030I, p.P1088S, and p.L1143P disrupt BRCA2-PALB2 interaction; p.W912G, p.G937R, p.I944N, p.L961P, p.L972Q, p.T1030I, p.I1037T, p.G1043D, and p.L1172P are associated with PALB2 protein instability; and p.I944N, p.L947F, p.L947S, p.T1030I, p.L1070P, and p.W1140G result in the mislocalization of PALB2 to the cytoplasm. However, p.L35P remains the only recognized *PALB2* pathogenic missense variant validated by systematic *in vitro* functional assays. Further functional analysis and people-based screening data are needed to properly evaluate the pathogenicity of *PALB2* VUSs.

## Conclusions and Perspectives

To date, the specific structures, multifaceted functions, and complex regulatory networks of PALB2 have been elaborated by multiple studies. PALB2 is a crucial regulator in maintaining genome integrity, while its dysfunction leads to breast cancer predisposition. The clinical relevance of PALB2 has been partially described, and *PALB2* is reported to be a high-risk breast cancer susceptibility gene comparable to *BRCA2* (16). With the identification of deleterious *PALB2* recurrent mutations and PARPi, individualized risk assessment and precision medicine for *PALB2* mutation-associated breast cancer become possible. Nevertheless, caution is warranted before promoting specific treatments, such as preventive surgery, as the existing experimental and clinical data are not sufficient. Moreover, plenty of *PALB2* VUSs emerged in large-scale *PALB2* screenings; however, their pathogenicity remains undefined, thereby precluding their clinical application. Altogether, to deliver individualized precision medicine, further long-term, population-based *PALB2* mutation studies combined with systematic functional verification are required.

## Author Contributions

YC and SW designed the subject of the review. SW, JZ, and KZ wrote the manuscript. SW, HC, ML, YL, and YS compiled the figures. JZ and YC reviewed the manuscript. All the authors read and approved the final manuscript.

### Conflict of Interest

The authors declare that the research was conducted in the absence of any commercial or financial relationships that could be construed as a potential conflict of interest.

## Abbreviations

ATM: ataxia telangiectasia mutated protein
ATR: ATM and Rad3-related kinase
CDK: cyclin-dependent kinase
ChAM: chromatin-association motif
CI: confidence interval
DSBs: DNA double-strand breaks
FA: Fanconi anemia
FPC: familial pancreatic cancer
H3K36me3: lysine 36-trimethylated histone H3
HR: homologous recombination
MBC: male breast cancer
MMC: mitomycin C
MRN: Mre11–RAD50–Nbs1 complex
NES: nuclear export sequence
OR: odds ratio
PALB2: partner and localizer of BRCA2
PARP: poly (ADP-ribose) polymerase
PARPi: PARP inhibitor
PV: pathogenic variant
ROS: reactive oxygen species
RPA: replication protein A
SETD2: SET domain containing 2
SSA: single-strand annealing
ssDNA: single-stranded DNA
VUSs: variants of unknown significance.
